# Colon angiolipoma with intussusception: a case report and literature review

**DOI:** 10.1186/1477-7819-11-69

**Published:** 2013-03-15

**Authors:** Lei Wang, Ping Chen, Liang Zong, Guang-Yao Wang, Hao Wang

**Affiliations:** 1 Department of Gastrointestinal Surgery, Su Bei People’s Hospital of Jiangsu Province, Yangzhou University, Yangzhou 225001, Jiangsu Province, China

**Keywords:** Gastrointestinal tract angiolipoma, Colon angiolipoma, Intussusception, Colon cancer

## Abstract

Angiolipomas are frequently observed benign tumors. They have a typical vascular component and are often located in subcutaneous tissues, and more rarely, in the gastrointestinal tract. We present the case of a 58-year-old man who complained of abdominal discomfort in the left lower quadrant and two to three bloody stools a day without any obvious etiology. These symptom became more severe in the next three days, due to a large angiolipoma located in the descending colon, which was diagnosed intraoperatively. In a literature review, we found only 22 cases of angiolipomas involving the gastrointestinal tract which are reported in the literature from 1960 to 2012 in PubMed; the key words used in the search are gastrointestinal tract angiolipoma, esophagus, stomach, duodenum, intestine, ileocecal junction, colon, rectum angiolipomas. Colon angiolipoma with intussusception, as seen in this case, is rare and may require emergent surgical intervention.

## Background

Angiolipoma is a benign tumor, commonly occurring in the subcutaneous tissue and other locations, but is rarely found in the gastrointestinal tract. Histologically, it is comprised of adipose tissue and proliferative blood vessels and is usually diagnosed postoperatively. This report focuses on the importance of correct pre- and/or intraoperative histological diagnosis in order to offer the best therapeutic choice with the diagnosis confirmed postoperatively.

## Case presentation

**Figure 1:**
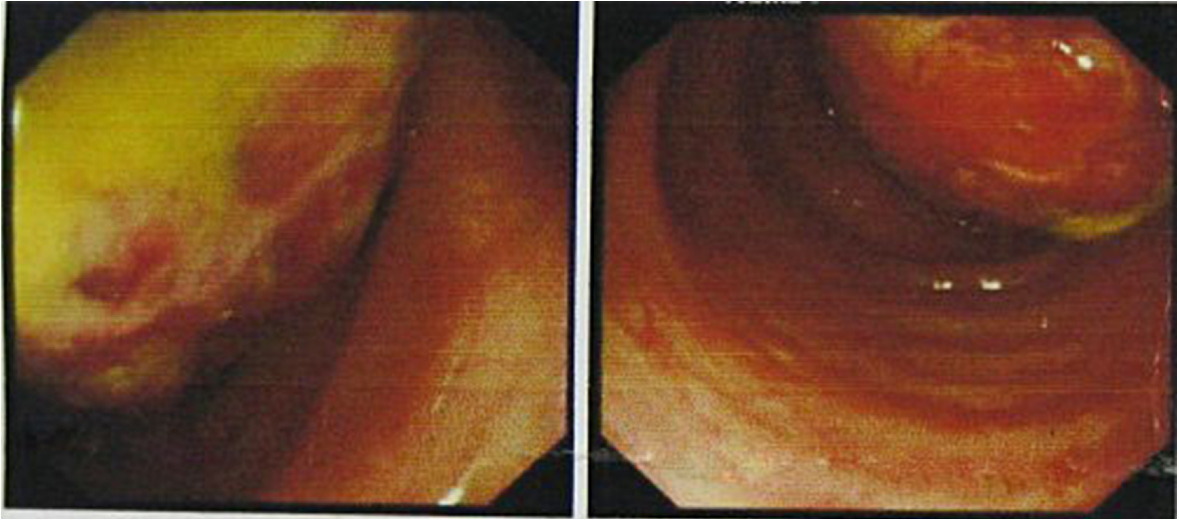
**Colonoscopy revealed a large pedunculated, cauliflower shaped mass with superficial ulceration and necrosis at approximately 40 cm from the anal verge.** The mass appeared hard to the touch and friable.

**Figure 2:**
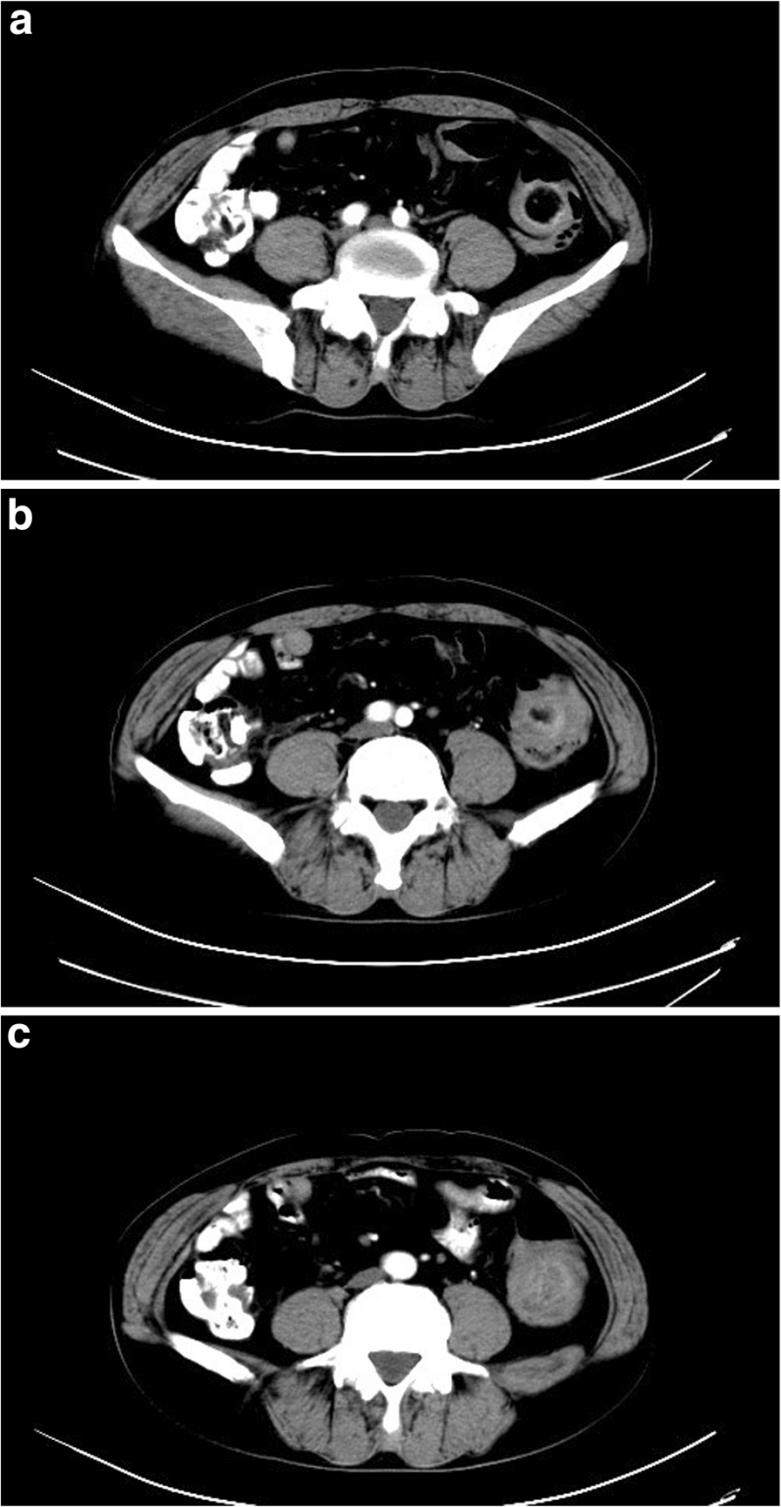
**Abdominal CT scan with contrast from (a) to (c) showed a 5 cm diameter mass-like lesion at the junction of the descending and sigmoid colon with significantly thickened walls with dilated lumen.** There were multiple rings within the mass with smooth margins suggesting colon intussusception. CT, computed tomography.

**Figure 3:**
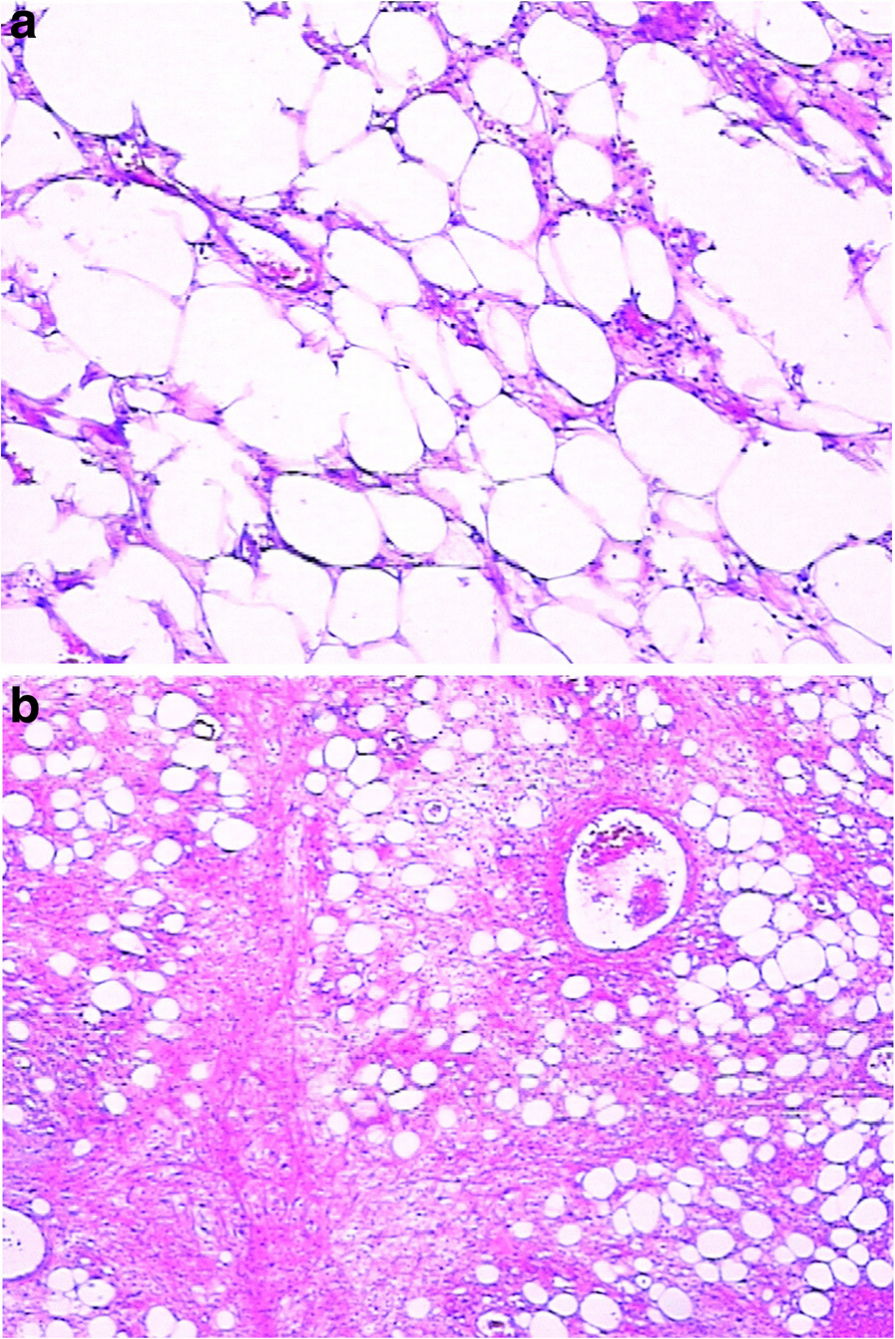
Histologic examination from (a) and (b) revealed a mass consisting of mature adipose tissue with vascular structures.

A 58-year-old male patient was admitted to Su Bei People’s Hospital of Jiangsu Province on 13 March 2012, with a major complaint of ‘lower left quadrant abdominal discomfort with bloody stools.’ His symptoms began about a month prior to the abdominal discomfort in the lower left quadrant and he experienced two to three bloody bowel movements a day without an obvious etiology. These symptoms became more severe in the next three days. Blood appeared purple and red on the surface of soft stools combined with mucus. He also experienced occasional abdominal pain and tenesmus. Colonoscopy (Figure 1) at an outside hospital on 5 March 2012 had revealed a large pedunculated, cauliflower shaped lump with superficial ulceration and necrosis at approximately 40 cm from the anal verge. The mass appeared hard and friable. The rest of the colon and rectum appeared normal. Biopsy of the lesion showed non-specific chronic mucosal inflammation with necrosis and exudate. The patient’s past medical history and family history were unremarkable. At his physical examination on arrival at our hospital, the abdomen was soft, non-distended and without visible peristalsis. There was mild tenderness on palpation without rebound tenderness. No lumps were spotted. Rectal examination was unremarkable and blood was present. Laboratory tests showed a white blood cell count of 7.4 × 10^9^/L, a red blood cell count of 3.08 × 10^12^/L, a neutrophil count of 5.78 × 10^9^/L (78.1%), and a hemoglobin level of 90 g/L. Tumor markers were as follows: alpha fetoprotein (AFP) 3.87, CA 199, and carcinoembryonic antigen (CEA) 2.29. An abdominal CT scan with contrast showed a 5-cm diameter lump-like lesion at the junction of the descending and sigmoid colon with significantly thickened walls with dilated lumen (Figure 2). There were multiple rings within the lump with smooth rims suggesting colon intussusception. Colon cancer could not be ruled out. The patient was urgently taken to the operating room to probe with a preoperative diagnosis of colon intussusception and possible colon cancer. Intraoperatively, the mass was located at the splenic flexure measuring 10 × 7 cm. Colon intussusception was present with dilatation of the proximal colon. No other lesions were identified. Once the intussusception was reduced, an 8 × 5 cm colon lesion was revealed and a partial colectomy was performed. Frozen section revealed an angiolipoma. No further resection was indicated. The patient recovered from surgery well and was taken back to the ward. Final pathology showed an 8 × 6 × 6 cm exophytic colon lesion consisting mainly of fatty tissue with ulceration. Seven lymph nodes ranging from 2 mm to 10 mm were identified outside the colon. Histologic examination (Figure 3) revealed a mass consisting of mature adipose tissue with vascular structures. Intralesional fat necrosis and mucosal ulcerations were present with a large amount of neutrophil and lymphocyte infiltration. Lymph nodes showed reactive proliferation with no evidence of tumor. The final pathologic diagnosis was angiolipoma of the descending colon. During follow-up, this patient showed no recurrence.

## Discussion

Angiolipoma was first described in 1912 by Bowen [1]. In 1960, Howard [2] demonstrated that the clinicopathological features of angiolipomas differed from those of lipomas, thereby delineating angiolipoma as a new entity. It can be classified by the ratio of adipose and vascular tissue composition as predominantly lipomatous or angiomatous type [3]. Cytogenetic analysis shows the normal karyotype of angiolipomas contrasts with the various other types of benign lipomatous tumors, most of which show rather specific clonal chromosomal aberrations [4], which suggests that the pathogenesis of angiolipomas may be different from that of ordinary lipomas. Angiolipomas usually develop as encapsulated subcutaneous tumors, most commonly on the arms and trunk in young adults. They are rarely larger than 2 cm in diameter, frequently multiple in number and characteristically tender or painful [5]. They are rarely found in the gastrointestinal tract. In a literature review, we found only 22 cases of angiolipomas involving the gastrointestinal tract. Of these, one was located in the esophagus [6], three in the stomach [7-9], two in the duodenum [10,11], six in the small intestine [12-17], three at the ileocecal junction [18-20], five in the colon [3,10,21-23], and two in the rectum [24,25]. Angiolipomas in the gastrointestinal tract usually do not have specific clinical manifestations. Patients are often asymptomatic when the tumor is small. With increasing size of the tumor, they may experience abdominal pain, abdominal distension, melena, and symptoms of intussusception and bowel obstruction. Colon angiolipoma with intussusception, as seen in this case, is rare and may require urgent surgical intervention. Only two such cases have been reported in the literature [23,26].

Radiological examination including barium radiographs, enteroclysis, abdominal ultrasound, computed tomography (CT), and magnetic resonance imaging (MRI) can detect the lesion in the gastrointestinal tract before histopathological diagnosis. Barium enema [3] and enteroclysis [17] may show a filling-defect in the lumen which usually appears as a hyperechoic lesion on transabdominal ultrasonography [3,16] and a submucosal lesion in the gastrointestinal wall in endosonography [6] with central high signal intensity and peripheral iso-signal intensity on T1-weighted inphase images [17]. CT image characteristics depend on the tissue composition of the lesion, from a lipomatous type comprised of fat without contrast enhancement, which is often diagnosed as a lipoma [27], to a low-fat containing tumor showing numerous small round density enhancements [28,29]. In 1998, Mintz and Mengoni [30] reported the use of sonography to diagnose a breast angiolipoma, which appeared as a homogeneously hyperechoic density on echogram. Chen *et al*. [3] also reported the successful use of imaging to diagnose an angiolipoma that was later confirmed with surgical pathology. However, the overall pre-operative diagnostic accuracy for gastrointestinal angiolipomas is quite low due to the non-specific clinical presentations and lack of specific findings on imaging studies. Error in diagnosis can lead to inappropriate surgery such as an unnecessary radical resection because of an erroneous preoperative diagnosis of colon cancer. The correct diagnosis of gastrointestinal angiolipomas is usually made intraoperatively and confirmed by final surgical pathology. When possible, an intraoperative biopsy with frozen section may provide an accurate diagnosis to guide surgery. In our case, preoperative biopsy during colonoscopy only showed non-specific inflammation without tumor cells. Combined with the CT findings, we doubted the presence of colon cancer and elected to obtain intraoperative frozen sections, which indeed revealed a benign process, thus avoiding an unnecessary radical resection.

The treatment options for colon angiolipoma vary depending on the type of lesions. Small pedunculated polyps can be removed under colonoscopy. Injection of epinephrine,and the use of nylon loop or metal hemostatic clips prior to polypectomy may effectively decrease intraoperative bleeding [10,22]. For large lesions or broad-based polyps, surgical excision is the treatment of choice. Compared to endoscopic excision, surgical resection may reduce the risk of perforation and hemorrhage in these patients [3]. However, minimally invasive procedures such as laparoscopic resection may be possible. Vandamme *et al*. [21] reported a case of cecal angiolipoma that was successfully treated with laparoscopy-assisted ileocecostomy and a five-year follow-up showed no recurrence. Ishizuka *et al*. [25] successfully treated a rectal angiolipoma through a transanal approach. Open surgical resection was chosen for our patient due to the large size of the tumor (8 × 6 × 6 cm) and evidence of colon intussusception which required urgent examination. Because angiolipomas are benign tumors, only limited colon resection should be performed provided that the tumor can be completely removed [19,22]. Studies have shown [12,25] that excellent prognosis can be expected with complete tumor removal although recurrence is high when the tumor is inadequately resected.

## Conclusion

Angiolipoma is a benign tumor, commonly occurring in the subcutaneous tissue and is rarely found in the gastrointestinal tract. Error in diagnosis can lead to inappropriate surgery such as an unnecessary radical resection because of an erroneous preoperative diagnosis of colon cancer. We should focuses on the importance of correct pre- and/or intraoperative histological diagnosis in order to offer the best therapeutic choice.

## Consent

Written informed consent was obtained from the patient for publication of this case report and any accompanying images.

## Competing interests

The authors declare that they have no competing interests.

## Authors’ contributions

WL and CP performed the majority of this study and wrote the manuscript; ZL, WGY and WH provided the collection of material from the database. All authors read and approved the final manuscript.
